# Polymorphisms in Telomere Length Associated *TERC* and *TERT* predispose for Ischemic Stroke in a Chinese Han population

**DOI:** 10.1038/srep40151

**Published:** 2017-01-06

**Authors:** Shuo Zhang, Guofa Ji, Yiqian Liang, Rui Zhang, Puyu Shi, Dangshe Guo, Chunqi Li, Jing Feng, Feng Liu, Rong Peng, Mingwei Chen

**Affiliations:** 1 Department of Respiratory and Critical Care Medicine, The First Affiliated Hospital of Xi’an Jiaotong University, Xi’an 710061, P. R. China; 2 Shaanxi Provincial Research Center for the Project of Prevention and Treatment of Respiratory Diseases, Xi’an 710061, P. R. China; 3 Department of Respiratory Medicine, Xi’an 141 Hospital, Xi’an 710089, P. R. China; 4 Department of Respiratory Medicine, Hospital of Lantian County, Xi’an 710500, P.R. China; 5 Department of Internal Medicine, Shaanxi Normal University Hospital, Xi’an 710062, P. R. China; 6 Department of Internal Medicine, Xi’an International Studies University Hospital, Xi’an 710061, P. R. China; 7 Medical Information Management Office, The First Affiliated Hospital of Xi’an Jiaotong University, Xi’an 710061, P. R. China

## Abstract

The role of telomere in genomic stability is an established fact. Variation in leukocyte telomere length (LTL) has been considered a crucial factor that associated with age-associated diseases. To elucidate the association between LTL variation and ischemic stroke (IS) risk, we selected ten single nucleotide polymorphisms (SNPs) in three genes (*TERC, TERT* and *RTEL1*) that previously reported link to LTL, and genotyped SNPs of these genes in a case-control study. The association between polymorphisms and IS risk were tested by Chi squared test and haplotype analysis. In allele association analysis, allele “C” in rs10936599 of *TERC* gene and allele “G” in rs2853677 of *TERT* gene were found to have an increased risk of IS when compared with allele “T” and “A”, respectively. Model association analysis showed that genotype “G/A” in the overdominant model and genotypes “G/A” and “A/A” in the dominant model of rs2242652 presented a more likelihood to have IS. Another *TERT* locus (rs2853677) with genotype “G” was also found IS-related risky in the log-additive model. Taken together, our results suggest a potential association between LTL related *TERC, TERT* gene variants and ischemic stroke risk.

Ischemic stroke (IS) presents a leading cause of persistent and acquired adult disability^1^. An unprecedented 50% increase in stroke incidence has been predicted to China for the next 20 years based on the current demographic and population-level vascular risk factor trends^2^. In spite of recent development in therapy, ischemic stroke patients still suffer a dismal prognosis, which in part is due to indefinite etiology and pathogenesis^3^. Smoking, arterial hypertension, diabetes mellitus, dyslipidemia, adiposity and alcohol have been verified attributive factors to the increased risk of ischemic stroke^4^,^5^,^6^,^7^. However, there tend to be more and more patients diagnosed with stroke in the absence of the above factors. Genetic susceptibility to stroke independent of the distinct risk factors has not yet been clearly validated. Thus, we hypothesize that the stroke risk may be caused by the variation in low-penetrance alleles.

Telomerase is a ribonucleoprotein polymerase that prolongs leukocyte telomere length (LTL) by adding a repetitive sequence “TTAGGG” in humans to antagonize DNA polymerase inability of fully replicating the 3’ end of DNA strand in mitotic division^8^,^9^,^10^. Variation in LTL has been found among individuals with same gender and age and has been increasingly accepted as an important factor of many age-associated diseases, such as ischemic stroke^11^,^12^,^13^.

In this study, we selected single nucleotide polymorphisms (SNPs) from three different LTL related genes *(TERC (telomerase RNA component), TERT (telomerase reverse transcriptase) and RTEL1 (regulator of telomere elongation helicase 1))* that have been linked to ischemic stroke^14^,^15^,^16^,^17^,^18^,^19^. We analyzed each tag single nucleotide polymorphism (tSNP) in a case-control study involving Chinese population. Chi squared test and haplotype analysis were used to test the association between gene polymorphisms and ischemic stroke risk. *TERC* and *TERT* variants were found linked to ischemic stroke risk.

## Results

We recruited all together 300 ischemic stroke patients (120 females and 180 males, mean age at diagnosis 60.3 years, range 51–69, SD ± 5.14) and 300 healthy individuals (120 females and 180 males, mean age at 60.4 years, range 54–78, SD ± 6.36) to our study [Table 1]. None of the tSNPs we evaluated among the control group deviated from HWE [Table 2]. We hypothesized that the minor allele of each SNP was the risk factor as compared to the wild-type allele.

The results indicated two significant aggressive alleles, “C” in rs10936599 of *TERC* gene and “G” in rs2853677 of *TERT* gene, were linked to an increased ischemic stroke risk based on the *P* value of 0.05 (OR = 1.26; 95% CI, 1.00–1.58; *P* = 0.049 and OR = 1.35; 95% CI, 1.07–1.71; *P* = 0.012, respectively) by Chi-square test [Table 2].

Subsequent various genetic models were performed to calculate the genetic risk. The data showed that in rs2242652 of *TERT* gene, an enhanced risk of ischemic stroke was associated with the genotype “G/A” in the over-dominant model (OR = 1.45, 95% CI, 1.02–1.77; *P* = 0.038) and genotypes “G/A” and “A/A” in the dominant model (OR = 1.42, 95% CI, 1.01–2.01; *P* = 0.044) [Table 3]. As for rs2853677 listed in Table 4, patients who carried genotype “G” were also found to have a more likelihood of suffering ischemic stroke in the log-additive model (OR = 1.36, 95% CI, 1.07–1.73; *p* = 0.011) after adjustment by gender and age.

However, after performing a strict Bonferroni correction in Tables 2, 3 and 4, the significance levels were attenuated, which may indicate a likely association between positive tSNPs and risk of IS.

We then did Haplotype analysis to explore the connection between the *TERT, RTEL1* haplotype and the risk of ischemic stroke, but no significant conclusion was found [Tables S1, S2].

## Discussion

In this case-control study, we selected tSNPs with MAF greater than 5% in the HapMap CHB population to ensure the sufficient statistical power for data analysis. Our results revealed that polymorphisms in *TERC* and *TERT* genes have an association with susceptibility risk of ischemic stroke in a Chinese Han population. We hypothesize that these loci variant of the *TERC* and *TERT* genes could have shortened LTL, leading to increased possibility of having ischemic stroke.

The *TERC* gene, one of the main components of telomerase, serves as a template for addition of multiple “TTAGGG” repeats^9^,^20^. It has been verified involved in LTL by recent researches^21^,^22^. We found in this study that the allele “C” in rs10936599 had the potential to increase the risk of ischemic stroke when compared with allele “T” (OR = 1.26; 95% CI, 1.00–1.58; *P* = 0.049). Studies focused on similar subject by Zee RY, *et al*. involving Caucasian women did not find any association between *TERC* and ischemic stroke^23^. We believe this disparity in findings could be attributed to the racial, sexual or regional differences in study populations. To our knowledge, our study is the first genotype/allele-based study that describes the association between SNPs within the *TERC* locus and ischemic stroke risk in a Chinese population.

The *TERT* gene, located in 5p15.33, encodes the catalytic protein component of telomerase, which is required for maintenance of LTL, chromosomal stability and cellular immortality^24^,^25^. Mutations occur in the *TERT* gene can shorten LTL and are major risk factors for stroke^26^,^27^,^28^, also for multiple cancers^25^ and other syndromes, including idiopathic pulmonary fibrosis and aplastic anemia^29^. A recent study by Bresssler J, *et al*. found that rs2853668 of *TERT* was nominally associated with stroke (OR =  1.17, *p* =  0.05, 95% CI =  1.00–1.38) in African-Americans, but failed to draw the same conclusion in Caucasian study participants or with mortality in either racial group^26^. In this case-control study, we found that rs2853677, an intrinsic SNP within the *TERT* gene, was significantly associated with ischemic stroke risk according to both allele and genotype association analysis in a Chinese population. We ascertained a significant allele “G”, genotype “G/G” in the co-dominant model, genotypes “A/G” and “G/G” in the dominant model and genotype “G/G” in the recessive model aggressive for ischemic stroke development. Subsequent model association analysis of another locus within *TERT* gene, rs2242652, also found that genotype “G/A” in the over-dominant model and genotypes “G/A” and “A/A” in the dominant model increased the ischemic stroke risk. Previous studies about the loci above were mainly focused on multiple cancer risks^30^,^31^,^32^,^33^,^34^. Campa D, *et al*. identified a significant association between a variant in *TERT* and pancreatic cancer risk (rs2853677, odds ratio =  0.85; 95% confidence interval =  0.80–0.90, *P* =  8.3 × 10^−8^)^35^. Our study, for the first time, combined rs2853677 and rs2242652 polymorphisms with ischemic stroke risk. All these results together with previous studies strongly implicate the involvement of *TERT* in ischemic stroke.

There are certain intrinsic limitations in our study and must be noted. The sample size was not large enough compared with some other ischemic stroke association studies. Further work with larger sample size is needed to consolidate our conclusion. We performed Bonferroni correction in statistical analysis and found the significance levels between *TERT* and *TERC* SNPs and IS risk were attenuated. Such issue might partly due to the relative small sample size that could not satisfy all of the ten independent hypotheses at the same time. Moreover, we could not ignore the main weakness of Bonferroni correction. True important differences may tend to be deemed nonsignificant as the times of tests performed in one study increase. As a result, more type II errors accurs^36^. Cumulatively, our findings provide evidence that polymorphisms in *TERC* and *TERT* genes variation are associated with increased ischemic stroke risk. We believe our results will encourage further studies to explore the functional role of these genes.

## Methods

### Study participants

A case-control study containing 300 ischemic stroke patients and 300 controls was conducted at the First Affiliated Hospital of Xi’an Jiaotong University. All the patients we recruited were newly diagnosed of ischemic stroke at Neurology Department from the year 2014 to 2015 and all the control subjects were enrolled from the health check-up center for annual health examination. The diagnostic criteria for ischemic stroke were based on the International Classification of Diseases (9th Revision). Related clinical and demographic data of the case and control groups were collected by medical record and face-to-face questionnaires. All of the participants were genetically unrelated ethnic Han Chinese. They were all provided with written informed consent for their participation and the informed consent was obtained from all subjects in the present study. The protocols for this study were conducted according to the Declaration of Helsinki and were approved by the Institutional Review Boards of both the First Affiliated Hospital of Xi’an Jiaotong University and Northwest University. 5 mL whole blood of each subject was extracted at the time of initial diagnosis. The samples were stored at −80 °C until further use.

### tSNP selection and genotyping

The minor allele frequencies (MAF) of all the selected SNPs were greater than 5% in the HapMap CHB (Chinese Han Beijing) population. DNA was extracted from whole blood using GoldMag-Mini Whole Blood Genomic DNA Purification Kit (GoldMag Co. Ltd. Xi’an City, China). DNA concentration was measured by NanoDrop 2000 (Thermo Scientific, Waltham, Massachusetts, USA). The design of primers, SNP genotyping and data processing were performed by Sequenom MassARRAY platform Software (Sequenom Co. Ltd., San Diego, California, USA)^37^,^38^. Genotype calling was carried out with 3.0.0.4 version MassARRAY RT software and analyzed by 3.4 version MassARRAY Typer software^37^,^39^,^40^. Genotyping quality control procedures leading to SNP exclusion were call rate < 90% and *P* < 0.05 for deviations from HWE. All of the selected SNPs in the study were successfully genotyped with average call rate of 99.68%.

### Statistical analysis

Statistical analysis was performed using statistical software (SPSS 18.0; Chicago, IL) and Microsoft Excel. Statistical significance was accepted at *P* value < 0.05. Hardy-Weinberg equilibrium (HWE) of each tSNP in control group was tested by Fisher’s exact test. The differences between allelic frequencies in case and control groups were compared by the Chi-squared test^41^. Association between genotypes and ischemic stroke risk were estimated in different genetic models (co-dominant, dominant, recessive, over-dominant and log-additive) by SNPStats website software http://bioinfo.iconcologia.net/snpstats/start.htm^42^. Testing of odds ratios (ORs) and 95% confidence intervals (CIs) were performed by unconditional logistic regression analysis adjusted by gender and age^43^. Akaike’s Information Criterion and Bayesian Information Criterion were applied to estimate the best-fit model for each SNP.

## Additional Information

**How to cite this article**: Zhang, S. *et al*. Polymorphisms in Telomere Length Associated *TERC* and *TERT* predispose for Ischemic Stroke in a Chinese Han population. *Sci. Rep.* **7**, 40151; doi: 10.1038/srep40151 (2017).

**Publisher's note:** Springer Nature remains neutral with regard to jurisdictional claims in published maps and institutional affiliations.

## Supplementary Material

Supplementary Information

## Figures and Tables

**Table 1:** Characteristics of patients and controls.

Characteristics	Ischemic stroke (n = 300)		Control (n = 300)		*P* value^a^
Age (means ± SD, year)	60.3 ± 6.4		60.4 ± 5.1		0.849
Gender	No.	%	No.	%	
Male	180	60	180	60	1.000
Female	120	40	120	40	

^a^*P* value was estimated by Welch’s *t* test and Pearson’s *χ*^2^ test for age and gender variables, respectively. Abbreviation: SD, standard deviation.

**Table 2:** Candidate tag single nucleotide polymorphisms.

SNP ID	Gene name	Chromosome position	Position	Allele	Minor allele	MAF (Case)	MAF (Control)	*p*-value for HWE	ORs	95% CI		*P*	*P*^*a*^
rs35073794	TERC	3q26.2	169482135	A/G	A	0.008	0.007	1.000	1.25	0.33	4.69	0.738	1
rs10936599	TERC	3q26.2	169492101	C/T	C	0.482	0.425	0.906	1.26	1.00	1.58	0.049^*^	0.49
rs10069690	TERT	5p15.33	1279790	T/C	T	0.175	0.144	0.347	1.27	0.93	1.73	0.138	1
rs2242652	TERT	5p15.33	1280028	A/G	A	0.198	0.160	0.523	1.30	0.97	1.75	0.083	0.83
rs2853677	TERT	5p15.33	1287194	G/A	G	0.402	0.332	0.696	1.35	1.07	1.71	0.012^*^	0.12
rs2853676	TERT	5p15.33	1288547	T/C	T	0.188	0.147	0.817	1.35	1.00	1.83	0.053	0.53
rs6089953	RTEL1	20q13.33	62291008	G/A	G	0.273	0.253	0.762	1.11	0.86	1.44	0.429	1
rs6010621	RTEL1	20q13.33	62310872	G/T	G	0.265	0.242	0.753	1.13	0.87	1.46	0.370	1
rs4809324	RTEL1	20q13.33	62318220	C/T	C	0.088	0.092	1.000	0.96	0.65	1.43	0.840	1
rs2297441	RTEL1	20q13.33	62327582	A/G	A	0.333	0.295	0.331	1.19	0.94	1.53	0.154	1

Abbreviations: SNP, single nucleotide polymorphism; MAF, minor allele frequency; HWE, Hardy-Weinberg equilibrium; OR, odds ratio; CI, confidence interval.

^*^P < 0.05 indicates statistical significance.

^a^*P* value was adjusted by Bonferroni correction.

**Table 3:** Relationship between rs2242652 in *telomerase reverse transcriptase* gene and ischemic stroke risk (adjusted by gender and age).

Model	Genotype	Control (N, %)	Case (N, %)	OR (95% CI)	*P*	*P*^*a*^	AIC	BIC
Codominant	G/G	213 (71%)	190 (63.3%)	1.00	0.11	1	837.4	859.4
	G/A	78 (26%)	101 (33.7%)	1.46 (1.02–2.08)				
	A/A	9 (3%)	9 (3%)	1.12 (0.44-2.88)				
	G/G	213 (71%)	190 (63.3%)	1.00				
Dominant	G/A-A/A	87 (29%)	110 (36.7%)	1.42 (1.01–2.01)	0.044^*^	0.44	835.7	853.3
	G/G-G/A	291 (97%)	291 (97%)	1.00				
Recessive	A/A	9 (3%)	9 (3%)	1.00 (0.39–2.55)	1	1	839.7	857.3
	G/G-A/A	222 (74%)	199 (66.3%)	1.00				
Overdominant	G/A	78 (26%)	101 (33.7%)	1.45 (1.02–2.07)	0.038^*^	0.38	835.4	853
Log-additive				1.31 (0.97–1.77)	0.079	0.79	836.6	854.2

*p-value ≤ 0.05 indicates statistical significance.

Abbreviations: OR, odds ratio; CI, confidence interval; AIC, Akaike’s Information criterion; BIC, Bayesian Information criterion.

^a^*p* value was adjusted by Bonferroni correction.

**Table 4:** Relationship between rs2853677 in *telomerase reverse transcriptase* gene and ischemic stroke risk (adjusted by gender and age).

Model	Genotype	Control (N, %)	Case (N, %)	OR (95% CI)	*P*	*P*^*a*^	AIC	BIC
Codominant	A/A	132 (44%)	107 (35.7%)	1.00	0.038^*^	0.38	835.2	857.2
	A/G	137 (45.7%)	145 (48.3%)	1.31 (0.93–1.85)				
	G/G	31 (10.3%)	48 (16%)	1.91 (1.14–3.21)				
Dominant	A/A	132 (44%)	107 (35.7%)	1.00	0.036^*^	0.36	835.3	852.9
	A/G-G/G	168 (56%)	193 (64.3%)	1.42 (1.02–1.97)				
	A/A-A/G	269 (89.7%)	252 (84%)	1.00				
Recessive	G/G	31 (10.3%)	48 (16%)	1.65 (1.02–2.68)	0.04^*^	0.4	835.5	853.1
Overdominant	A/A-G/G	163 (54.3%)	155 (51.7%)	1.00	0.5	1	839.3	856.9
	A/G	137 (45.7%)	145 (48.3%)	1.12 (0.81–1.54)				
Log-additive				1.36 (1.07–1.73)	0.011^*^	0.11	833.3	850.9

*p-value ≤ 0.05 indicates statistical significance.

Abbreviations: OR, odds ratio; CI, confidence interval; AIC, Akaike’s Information criterion; BIC, Bayesian Information criterion.

^a^*p* value was adjusted by Bonferroni correction.
