# Supplementary material for: Polymorphisms in Telomere Length Associated TERC and TERT predispose for Ischemic Stroke in a Chinese Han population
Source: Sci Rep. 2017 Jan 6;7:40151. doi: 10.1038/srep40151 (PMC5216405; doi:10.1038/srep40151)
Supplement: Supplementary Information [file srep40151-s1.doc]

**Polymorphisms in Telomere Length Associated *TERC* and *TERT* predispose for** **Ischemic Stroke in a Chinese Han population**

Shuo Zhang^1, 2, *^, Guofa Ji^1, 2,3, *^, Yiqian Liang^1, 2^, Rui Zhang^1, 2,4^, Puyu Shi^1, 2^, Dangshe Guo^1, 2,5^, Chunqi Li^1, 2,6^, Jing Feng^1, 2^, Feng Liu^1, 2^, Rong Peng^7, #^, Mingwei Chen^1, 2, #^

^1^ Department of Respiratory and Critical Care Medicine, The First Affiliated Hospital of Xi'an Jiaotong University, Xi'an 710061, P. R. China;

^2^ Shaanxi Provincial Research Center for the Project of Prevention and Treatment of Respiratory Diseases, Xi'an 710061, P. R. China;

^3^ Department of Respiratory Medicine, Xi'an 141 Hospital，Xi'an 710089, P. R. China;

^4^ Department of Respiratory Medicine, Hospital of Lantian County, Xi’an 710500, P.R. China;

^5^ Department of Internal Medicine, Shaanxi Normal University Hospital, Xi'an 710062, P. R. China;

^6^ Department of Internal Medicine, Xi'an International Studies University Hospital, Xi'an 710061, P. R. China;

^7^ Medical Information Management Office, The First Affiliated Hospital of Xi'an Jiaotong University, Xi'an 710061, P. R. China;

* Contributed equally to this work.

**#** Correspondence to: Rong Peng (RP), Medical Information Management Office, The First Affiliated Hospital of Xi'an Jiaotong University, 277 West Yanta Road, Xi'an 710061, P. R. China；and Mingwei Chen (MC), Department of Respiratory and Critical Care Medicine, The First Affiliated Hospital of Xi'an Jiaotong University, 277 West Yanta Road, Xi'an 710061, P. R. China.

Email: [395695324@qq.com](mailto:395695324@qq.com) (RP); [chenmingwei@mail.xjtu.edu.cn](mailto:chenmingwei@mail.xjtu.edu.cn) (MC);

**Table S1. The haplotypes of two SNPs (rs10069690 and rs2242652) and risk of** **ischemic stroke (adjusted by gender and age)**

| Haplotype | Freq. | Case, Control Ratio Counts | Case-Control  Frequencies | χ² | *p*^a^ | OR (95% CI) | *p***^b^** |
| --- | --- | --- | --- | --- | --- | --- | --- |
| CG | 0.813 | 474.8 : 125.2, 500.9 : 99.1 | 0.791, 0.835 | 3.725 | 0.054 | 1 | - |
| TA | 0.158 | 103.8 : 496.2, 85.4 : 514.6 | 0.173, 0.142 | 2.12 | 0.145 | 1.30 (0.95 - 1.77) | 0.1 |
| CA | 0.021 | 15.2 : 584.8, 10.6 : 589.4 | 0.025, 0.018 | 0.846 | 0.358 | 1.62 (0.70 - 3.72) | 0.26 |

*p*^a^ for Chi-square test. *p*^b^ for logistic regression adjusted by age and gender.

**Table S2. The haplotypes of three SNPs (rs6089953, rs6010621 and rs4809324) and risk of ischemic stroke (adjusted by gender and age)**

| Haplotype | Freq. | Case, Control Ratio Counts | Case-Control  Frequencies | χ² | *p*^a^ | OR (95% CI) | *p*^b^ |
| --- | --- | --- | --- | --- | --- | --- | --- |
| ATT | 0.734 | 432.9 : 165.1, 445.0 : 153.0 | 0.724, 0.744 | 0.622 | 0.4303 | 1 | - |
| GGT | 0.165 | 107.0 : 491.0, 90.0 : 508.0 | 0.179, 0.151 | 1.755 | 0.1853 | 1.25 (0.91 - 1.71) | 0.18 |
| GGC | 0.087 | 50.0 : 548.0, 54.0 : 544.0 | 0.084, 0.090 | 0.168 | 0.6822 | 0.94 (0.63 - 1.41) | 0.18 |
| GTT | 0.012 | 6.1 : 591.9, 8.0 : 590.0 | 0.010, 0.013 | 0.271 | 0.6029 | 0.94 (0.63 - 1.41) | 0.62 |

*p*^a^ for Chi-square test. *p*^b^ for logistic regression adjusted by age and gender.
